# Cancer-associated fibroblasts promote the survival of irradiated nasopharyngeal carcinoma cells via the NF-κB pathway

**DOI:** 10.1186/s13046-021-01878-x

**Published:** 2021-03-01

**Authors:** Weiqiang Huang, Longshan Zhang, Mi Yang, Xixi Wu, Xiaoqing Wang, Wenqi Huang, Lu Yuan, Hua Pan, Yin Wang, Zici Wang, Yuting Wu, Jihong Huang, Huazhen Liang, Shaoqun Li, Liwei Liao, Laiyu Liu, Jian Guan

**Affiliations:** 1 Department of Radiation Oncology, Nanfang Hospital, Southern Medical University, Guangzhou, Guangdong China; 2 Chronic Airways Diseases Laboratory, Department of Respiratory and Critical Care Medicine, Nanfang Hospital, Southern Medical University, Guangzhou, Guangdong China; 3 Department of Obstetrics and Gynecology, The First Affiliated Hospital of Guangzhou Medical University, Guangzhou, Guangdong China; 4 Department of Oncology, Maoming People’s Hospital, Maoming, Guangdong China; 5 Department of Radiation Oncology, Guangdong 999 Brain Hospital, Guangzhou, Guangdong China

**Keywords:** Nasopharyngeal carcinoma, Cancer-associated fibroblast, Irradiation, IL-8, NF-κB pathway, Tranilast

## Abstract

**Background:**

Irradiation has emerged as a valid tool for nasopharyngeal carcinoma (NPC) in situ treatment; however, NPC derived from tissues treated with irradiation is a main cause cancer-related death. The purpose of this study is to uncover the underlying mechanism regarding tumor growth after irradiation and provided potential therapeutic strategy.

**Methods:**

Fibroblasts were extracted from fresh NPC tissue and normal nasopharyngeal mucosa. Immunohistochemistry was conducted to measure the expression of α-SMA and FAP. Cytokines were detected by protein array chip and identified by real-time PCR. CCK-8 assay was used to detect cell proliferation. Radiation-resistant (IRR) 5-8F cell line was established and colony assay was performed to evaluate tumor cell growth after irradiation. Signaling pathways were acquired via gene set enrichment analysis (GSEA). Comet assay and γ-H2AX foci assay were used to measure DNA damage level. Protein expression was detected by western blot assay. In vivo experiment was performed subcutaneously.

**Results:**

We found that radiation-resistant NPC tissues were constantly infiltrated with a greater number of cancer-associated fibroblasts (CAFs) compared to radiosensitive NPC tissues. Further research revealed that CAFs induced the formation of radioresistance and promoted NPC cell survival following irradiation via the IL-8/NF-κB pathway to reduce irradiation-induced DNA damage. Treatment with Tranilast, a CAF inhibitor, restricted the survival of CAF-induced NPC cells and attenuated the of radioresistance properties.

**Conclusions:**

Together, these data demonstrate that CAFs can promote the survival of irradiated NPC cells via the NF-κB pathway and induce radioresistance that can be interrupted by Tranilast, suggesting the potential value of Tranilast in sensitizing NPC cells to irradiation.

**Supplementary Information:**

The online version contains supplementary material available at 10.1186/s13046-021-01878-x.

## Background

NPC is an Epstein-Barr virus (EBV)-associated cancer, prevalent in southeast Asia and north Africa [1]. According to the statistics published in 2018, cancer arising from the nasopharynx is responsible for 129,079 cases and 72,987 deaths each year [2]. Despite the early detection and advances in radiotherapy, treatment for patients who partially responded to irradiation continue to be unsatisfactory primarily due to the acquisition of radioresistance and tumor recurrence following irradiation [3, 4]. However, the underlying mechanisms remain elusive.

The tumor microenvironment (TME) consists of stromal cells, including fibroblasts, macrophages, endothelial cells, other immune cells, extracellular matrix, as well as the bioactive substances secreted by these cells [5]. As one of the most predominant stromal cell subtypes in solid tumors, cancer-associated fibroblasts (CAFs) contribute to most aspects of cancer progression, including proliferation, migration, invasion, angiogenesis, induction of chemoresistance and escape from immune-mediated killing [6–8]. It has been reported that CAFs can enhance cervical tumor growth after irradiation and promote the epithelial mesenchymal transition of pancreatic tumors via crosstalk between tumor cells and CAF-like vascular endothelial growth factors (VEGF) and fibroblast growth factors (FGF) [9, 10]. Radiobiological research has shown that the relationship between NPC and CAFs remains poorly understood. Recently, immune checkpoint inhibitors have emerged at the frontier in anticancer research for patients with recurrent or metastatic head and neck cancer following irradiation treatment [11, 12]. Although several breakthroughs have been made, the total efficacy of immunotherapy is finite. Tranilast is a CAF inhibitor [13] that has been reported to suppress CAF proliferation and inhibit the adverse effects induced by CAFs within the immune microenvironment [14]. For example, it has been reported that Tranilast treatment was associated with a reduced level of transforming growth factor-β1 production by CAFs. However, the precise mechanism by which Tranilast suppresses CAF activity in irradiation-related research remains unknown.

Our research has found that CAFs can activate the NF-κB pathway in irradiated NPC cells to reduce DNA damage, which could be interrupted by tranilast. These findings suggest that Tranilast may have potential value in increasing tumor sensitivity to irradiation. Thus, the latent function of Tranilast in NPC warrants further investigation in the future.

## Materials and methods

### Cell culture and regents

The human NPC cell lines, 5-8F, 6-10B, and HK-1 were donated by the Sun Yat-sen University Cancer Center. Cells were cultured in 1640 medium (Gibco) supplemented with 10% FBS (Gibco) and 1% penicillin/streptomycin (Thermo Scientific). Human CAFs and normal fibroblasts (NFs) were extracted from fresh NPC tissues and matched to normal nasopharynx tissues from patients diagnosed with local recurrence NPC patients treated with radical radiotherapy previously. Fibroblast isolation was performed as previously described [15]. Briefly, fresh tissues were cut into pieces of 2 to 3 mm and cultured in high-glucose DMEM medium (Gibco) for approximately one week until fibroblasts appeared. α-SMA was used to identify CAFs and NFs as described [6]. Specimens were obtained with written informed consent from patients with NPC enrolled at Nanfang Hospital, Southern Medical University. Characteristics of 4 CAF donors were documented in supplementary Table 1. In functional studies, NFs and CAFs were used within 10 passages. All cells were cultured in a humidified incubator containing 5% CO_2_ at 37 °C.

Human recombinant IL-8, HGF, and TNF-α were purchased from Peprotech (Suzhou, China). An antagonist of IL-8 receptor (Repertaxin), inhibitor of NF-κB activation (caffeic acid phenethyl ester [CAPE]), and a lactate inhibitor (sodium dichloroacetate, DCA) were purchased from Selleck Chemicals (Houston, USA); Tranilast was purchased from Target Mol (Massachusetts, USA).

### Establishment of radiation-resistant (IRR) 5-8F IRR cells

Establishment of IRR cell lines was performed as previously described [16]. In brief, cells were treated twice with irradiation at a graded dose of 2, 4, 6, 8 and 10 Gy, respectively. A total IR dose of 60 Gy was administered with the entire selection procedure. The final surviving 5-8F cells were verified, defined as radioresistant 5-8F cells, and termed 5-8F IRR. The parental 5-8F cells were treated without irradiation. Cells within 10 passages were used for experiments once the 5-8F-IRR cells were successfully established.

### Cell viability assay

CAFs were subjected to a cell viability assay using Cell Counting Kit-8 (Dojindo, Japan) according to the manufacturer’s instructions. In brief, CAFs were cultured in medium containing Tranilast at a concentration of 200 μM and 400 μM, respectively for two days and subsequently cultured at the density of 1.5 × 10^3^ cells per well in triplicate in 96-well plates containing Tranilast-free medium. After culturing for 8 h, CAFs were applied to CCK-8 assay and a subsequent detection was performed at the indicated time points. To detect the impact of Repertaxin and CAPE on NPC cell line proliferation, cells were cultured in medium containing a wide-range of reagent concentrations (0.01, 0.1, 1, 10, and 100 μM for CAPE and 1, 10, 40 μM for Repertaxin) for 2 - 4 days and then subjected to a CCK-8 assay.

### Colony forming assay

A total of 5-8F, 6-10B, and HK-1 cells were cultured in six-well plates for 12 h at various densities according to the aim of each experiment. The cell density was listed as follows: 300 (0 Gy), 400 (2 Gy), 1000 (4 Gy), 2000 (6 Gy), 4000 (8 Gy) per well for 5-8F and 6-10B and 500 (2 Gy), 10,000 (8 Gy) per well for HK-1. After a 2-week incubation, the six-well plates were obtained and colonies with more than 50 cells were counted. The surviving fraction (SF) was estimated using the following formula: SF = (number of colonies formed/number of cells seeded × plating efficiency of the control group), where plating efficiency was calculated as the ratio between colonies observed and the number of cells plated. Dose-response colony survival curves were plotted accordingly.

### Cytokine assay

CAF and NF cells were cultured in serum-free medium when grown to 80% confluence. After incubating for two days, the medium was collected and concentrated before dialysis. After labeling with biotin, the samples were applied to an antibody array chip (R&D; Cat ARY022B, LabEx) overnight. The following day, the chips were reacted with streptavidin-conjugated fluorescence dye, and detected with a chemiluminescence imager (Chemi Scope 6300). Data were normalized to the total protein.

### RNA sequencing and tissue microarray

Total RNA of parent 5-8F and 5-8F IRR cells were extracted using TrIzol reagent (Invitrogen Life Technologies, CA) according to the manufacturer’s instructions. The prepared RNA was then subjected to sequencing on a BGISEQ-500 platform at Beijing Genomics Institution (www.genomics.org.cn, BGI, Shenzhen, China). The level of gene expression was quantified and the NOISeq method was performed to screen for differentially expressed genes (DEGs) as previously described [17]. A tissue microarray was conducted by Shanghai Biotechnology Corporation using Affymetrix Genechip according to manufacturer’s protocol [18].

### Radiosensitivity index (RSI) analysis and gene set enrichment analysis

An RSI index served as an indicator for radiosensitivity, and was calculated as previously described [19]. In brief, head and neck squamous carcinoma (HNSCC) patients in the TCGA database and NPC patients in the GSE12542 dataset were divided into two groups based on the median of CAFs score using an online tool (https://gfellerlab.shinyapps.io/EPIC_1-1/). Following Racle’s study, 20 markers were used to identify CAFs [20]. Gene Set Enrichment Analysis (GSEA) software version 3.0 (Broad Institute, USA) was used to analyze GSE48501 and a human microarray containing radioresistant and radiosensitive NPC samples. A threshold of *P* ≤ 0.05 was applied for the analysis. Data for GSE48501 and GSE12542 were downloaded from the NCBI Gene Expression omnibus (GEO, http://www.ncbi.nlm.nih.gov/geo/).

### Lactate detection

Lactate was detected using a lactic acid measurement kit (Junji Biotechnology Co, China) according to the manufacturer’s protocol. Briefly, blank medium and conditioned medium produced by NFs and CAFs were prepared and subjected to a chromogenic reaction with the indicated reagents. The absorbance value was detected by a microplate reader machine (BIO-RAD 689). Data were normalized to the lactate standards.

### Comet assay

A comet assay was performed using a DNA Damage Detection Kit (Keygen, China). The cells were irradiated as described at dose of 2 Gy and treated in accordance with the experimental design. The following day, cells were harvested and suspended in PBS containing 1% low-melting agarose and layered onto adhesive microscope slides previously covered with 0.5% normal-melting agarose. The cells were dipped in a specific lysed buffer at 4 °C for 2 h. Next, the DNA was uncoiled and unwound in an alkalescent electrophoresis buffer for 30 min. Electrophoresis was performed and the cells were stained with 4′,6-diamidino-2-phenylindole (DAPI) solution for 10 min in a dark room. The slides were examined with an Eclipse fluorescence microscope (Nikon, Japan). Analysis of comet assay was performed as described [21].

### Real-time quantitative PCR

Total RNA was extracted from CAFs using TrIzol reagent (Invitrogen Life Technologies, CA) as instructed by the manufacturer’s protocol. SuperScript III First-Strand Synthesis SuperMix (Thermo Fisher Scientific, MA) was used for reverse transcription according to the manufacturer’s protocol. Real-time quantitative PCR (qRT-PCR) was implemented to measure specific mRNA expression using an ABI7500 FAST system with TaqMan Reverse Transcription Reagents and SYBR Green PCR MasterMix (Applied Biosystems, CA, USA). GAPDH was used as a loading control. The specific primers were listed as follows: GAPDH, forward: 5′ -GGAGCGAGATCCCTCCAAAAT-3′ and reverse: 5′-GGCTGTTGTCATACTTCTCATGG-3′. IL-8, forward: 5′-GTGCAGTTTTGCCAAGGAGT-3′ and reverse: 5′-CTCTGCACCCAGTTTTCCTT-3′. HGF: forward: 5′-TGGGCCATTCTATTCCCCC-3′ and reverse: 5′-CATGGGGTCAAGCTTCCAGT-3′. The △Ct expression values for amplification were calculated by normalizing to an internal control.

### Conditioned medium derived from human NFs and CAFs

CAFs and matched non-malignant NFs were cultured in 25 mL culture flask with high-glucose DMEM (Gibco) supplemented with 10% FBS at same density overnight, respectively. The cells were refreshed with serum-free DMEM and the supernatants were harvested after 48 h. Next, the cell pellet was removed after centrifugation and the conditioned medium (CM) was acquired and stored at − 80 °C for future experimentation.

### Western blotting analysis

Cells were lysed with RIPA lysis buffer (Beyotime, Shanghai, China) supplemented with phosphatase and a protease inhibitor cocktail (Roche). The proteins were subjected to 10% SDS-PAGE followed by transfer to PVDF membrane and subsequent incubation with specific primary antibodies at 4 °C overnight. Membranes were then incubated with respective secondary antibodies (FuDe, China) for 1 h at room temperature. The primary antibodies included mouse monoclonal anti-GAPDH (60004–1-IG, Proteintech, USA), rabbit monoclonal anti-α-SMA (ab124964, Abcam, USA), rabbit monoclonal p65 (8242, CST, USA), rabbit monoclonal p-p65 (3039, CST, USA), mouse monoclonal IKB-α (4841, CST, USA), and rabbit monoclonal γ-H2AX (9718, CST, USA). GAPDH was used as an internal reference.

### Immunofluorescence

Cells were seeded into a confocal dish and incubated overnight. The cells were washed three times with phosphate buffered saline (PBS) and fixed with 4% paraformaldehyde for 10 min. Next, 0.5% Triton X-100 was used for permeabilization and the samples were blocked with goat serum albumin. Subsequently, cells and tissues were incubated with primary antibodies targeted to α-SMA (1:400) and (or) rabbit monoclonal IL-8 (60004–1-IG, Proteintech, USA) at concentration of 1:100 or γ-H2AX (1:200) at 4 °C overnight, followed by an incubation with an anti-rabbit Alexa fluor-594-conjugated and (or) anti-rabbit Alexa fluor-488-conjugated secondary antibody, respectively. The nuclei were stained with 4′,6-diamidino-2-phenylindole (DAPI), and images were observed with an Eclipse fluorescence microscope (Nikon, Japan). Expression of α-SMA and IL-8 were determined by mean fluorescence intensity. Analysis of nuclear γ-H2AX foci was performed as described [21].

### IL-8 RNA knockdown in CAFs

A knockdown of IL-8 in CAFs was performed using small interfering RNA (siRNA) (RIBOBIO, China) with Lipofectamine 2000 (Invitrogen, CA) for 10 h of transfection. The specific sequence targeting IL-8 was listed as follows: GCCAAGGAGTGCTAAAGAA.

### Patients and clinical samples

All patients were treated with intensity-modulated radiation therapy with a radical dose ranging from 60 Gy to 70 Gy. Radioresistance was defined as the local relapse or recurrence of the ever irradiated area after 6 months of radiotherapy via enhanced computed tomography or enhanced magnetic resonance imaging [11]. Human NPC samples were obtained from Nanfang Hospital, Southern Medical University, including 6 cases of radioresistant tissues and 16 cases of radiosensitive tissues. The collection of clinical samples for research was approved by the Ethics Committee of the Nanfang Hospital.

### Immunohistochemistry assay

Each of the staining processes were conducted according to the manufacturer’s protocol. Primary antibodies consisted of rabbit monoclonal anti-α-SMA (ab124964, Abcam, USA) at a dilution of 1:400 and FAP (ab207178, Abcam, USA) at the dilution of 1:100. The levels of α-SMA and FAP expression were evaluated based on the staining intensity and percentage of positively-stained CAFs. Final scores were assessed as the total evaluation of staining intensity (0 for negative, 1 for weak, 2 for moderate and 3 strong) plus percent positivity (0 for 0%, 1 for 1% - 25%, 2 for 26% - 50%, 3 for > 50%) [22]. The IHC evaluation was performed by two independent pathologists [23].

### Mouse irradiation assays

Three-week-old male BALB/c nude mice were purchased from Southern Medical University Laboratory Animal Center (Guangzhou, China). Experiments involving animals were approved by the Institutional Animal Care and Use Committee of the Southern Medical University. Mouse models were generated by a subcutaneous inoculation of 5-8F cells alone or combined with human cancer-associated fibroblasts at a 1:1 ratio. The number of cancer cells in each injection was 1 × 10^6^ for one flank side. Mice were divided into six groups as required and received irradiation treatment at a 8Gy × 3 schedule 9 days after injection [24, 25]. Before irradiation, one co-injection group was subjected to an orthotopic injection of 200 μM Tranilast in 100 μL daily for three days [13]. The tumor volume was determined at the indicated time points with the following formula: tumor volume (mm^3^) = ½ × longest diameter 2 × shortest diameter. Mice were sacrificed and the tumors were excised after 2 weeks of the first irradiation. Treatment of irradiation was given at a dose rate of 500 cGy/min with a linear accelerator (Varian 2300EX, Varian, Palo Alto, CA) that generated 6MV X-ray.

### Statistical analysis

Statistical analysis of the data was performed using SPSS software version 20.0. Both paired and unpaired *t*-tests were performed to analyze the data between two experimental groups. Mann-Whitney tests were used to calculate the *P* values for IHC staining quantification. Data were presented as the means ± standard deviation (SD). Statistical significance was defined as a *P*-value less than 0.05. *P < 0.05; **P < 0.01; ***P < 0.001; ****P < 0.0001; ns, no significance.

## Results

### CAFs induce radioresistance of NPC cells

**Fig. 1:**
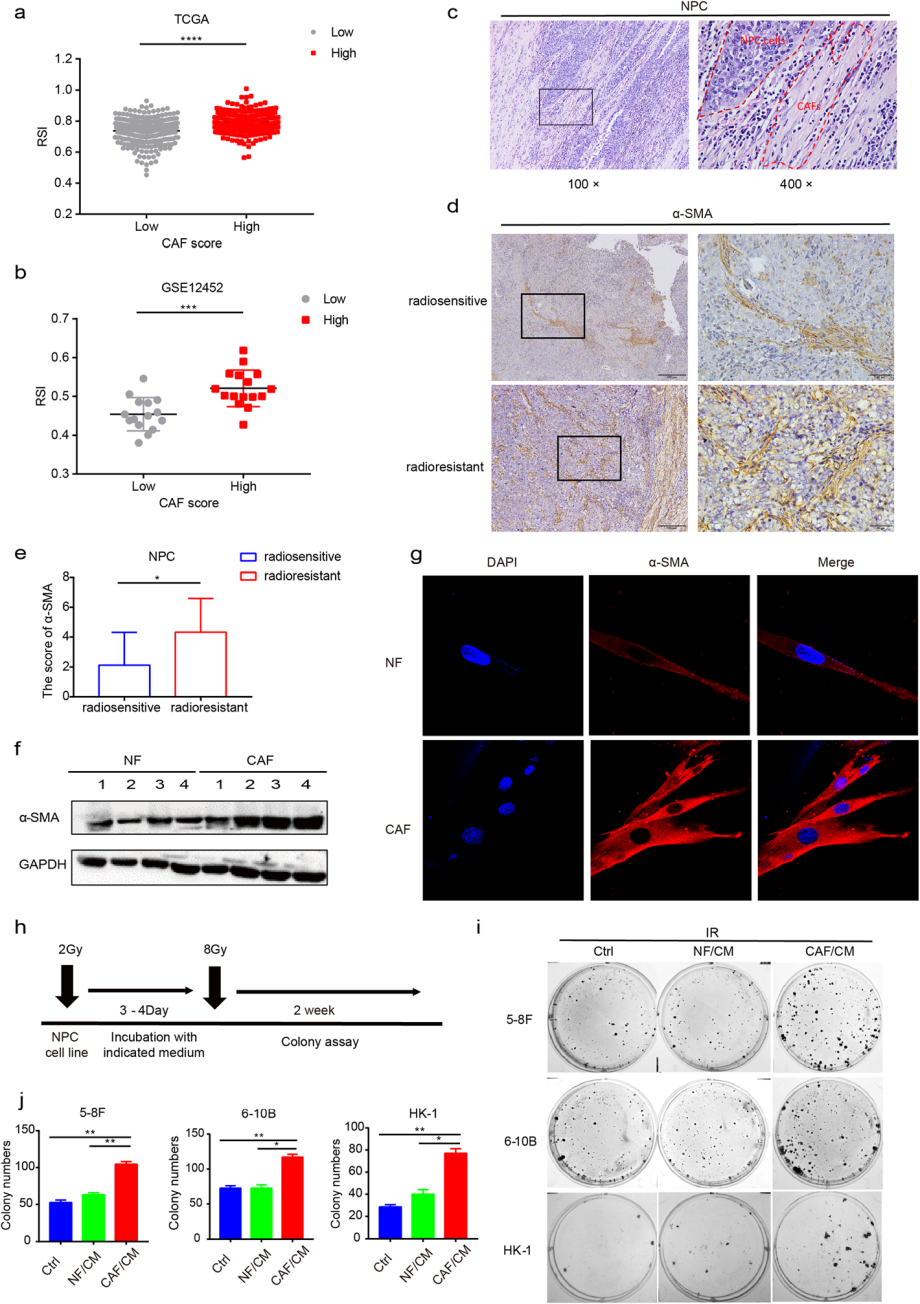
CAFs induced radioresistance of tumor cells. **a** and **b** RSI scores of tumors with abundant CAF infiltration were higher than the group with low CAF infiltration based on TCGA database and GSE12452 dataset, respectively. **c** HE staining showed the infiltration of CAFs in NPC tissue. **d** and **e** IHC showed greater CAF infiltration in radioresistant NPC tissue. **f** and **g** Western blot and immunofluorescence assay showed that CAFs expressed higher levels of α-SMA than NFs. **h**-**j** CAFs induced radioresistance of NPC cells. * *P* < 0.05; ** *P* < 0.01; *** *P* < 0.001; **** *P* < 0.0001, ns, no significance

Clinically, surgical resected radiation-resistant NPC tissues are stiff, which was also aligned with the findings of the physical examination, suggesting an abundance of stromal elements within the tissues. To uncover the role of CAFs in cancer irradiation treatment, patients with HNSCC in the TCGA dataset and NPC patients in the GSE12452 dataset were grouped based on the degree of CAF infiltration and the index of RSI was calculated. Consequently, patients with abundant CAF infiltration attained a higher RSI score (Fig. 1a and b), representing a lower sensitivity to irradiation. Furthermore, pathological analysis demonstrated that tissues from radiation-resistant carcinoma were infiltrated with substantial CAFs (Fig. 1c) that stained positive for α-SMA and FAP when compared with radiosensitive NPC (Fig. 1d and e; Fig. S1a and b). To date, little is known regarding the potential function of CAFs in assisting the acquisition of radioresistance for NPC. Thus, whether tumor cells surviving irradiation will acquire radioresistance characteristics following stimulation with CAF/CM remain unclear. To this end, we extracted and identified fibroblasts from the NPC tissues (Fig. 1f and g; Fig. S1c) and subsequently cultured the NPC cell lines with CM from CAFs or matched NFs following exposure of tumor cells to irradiation as illustrated (Fig. 1h). A colony assay demonstrated that the cells cultured with CAF/CM survived more the under higher given dose of 8Gy (Fig. 1i and j). These findings suggest that CAFs can induce radioresistance of NPC after adequate stimulation.

### CAFs secrete IL-8 to promote NPC cell survival upon irradiation exposure

**Fig. 2:**
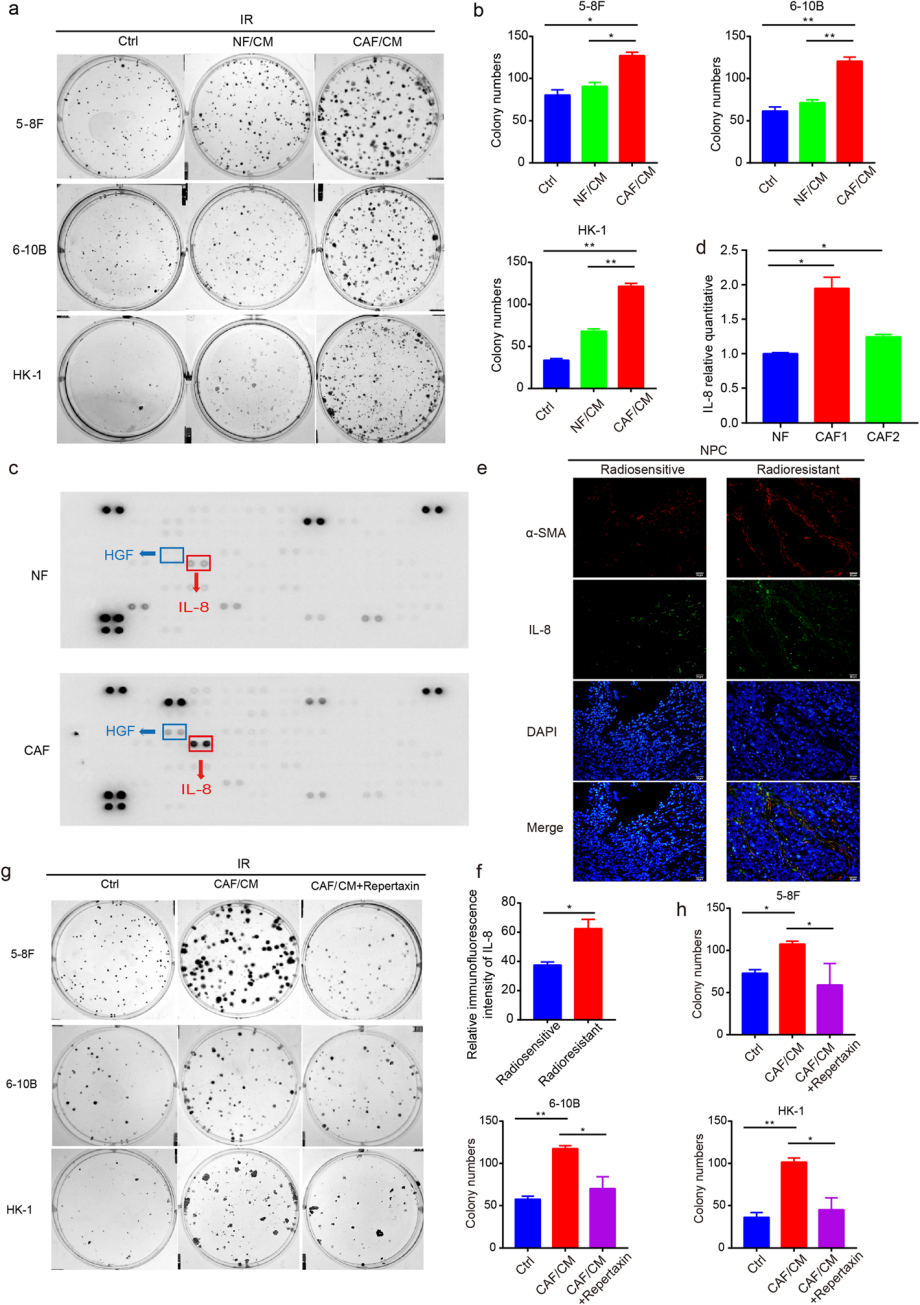
CAFs secreted higher level of IL-8 than NFs. **a** and **b** CAFs promoted survival of NPC cells after irradiation. **c** and **d** Higher level of IL-8 were detected in the CAF supernatants and evaluated by real-time PCR. **e** and **f** Represented Immunofluorescence images of IL-8 and α-SMA in NPC tissues were shown. **g** and **h** Treatment with an IL-8 receptor antagonist (10 μM) inhibited the proliferation of NPC cells after irradiation. * *P* < 0.05; ** *P* < 0.01; *** *P* < 0.001; **** *P* < 0.0001, ns, no significance

Next, we found that carcinoma cells supported with CAF/CM exhibited enhanced survival following 2Gy dose of irradiation compared with the group incubated with CM from NFs (Fig. 2a and b). Previous studies have suggested that CAFs secrete a variety of bioactive substances that contribute to tumor progression [26–28]. Moreover, CAFs were reported to produce a substantial amount of cytokines and generate an inflammatory environment for solid tumors [29]. Thus, a cytokine array was performed to understand the latent cytokines responsible for the enhanced survival and proliferation of irradiated NPC cells. By detecting CM production by CAFs, we found that interleukin 8 (IL-8) was substantially elevated compared with NF-derived CM, which was verified via RT-qPCR (Fig. 2c and d). Further study suggested that CAFs secreted IL-8 which was higher in radioresistant NPC tissues (Fig. 2e and f). Next, a functional assay showed that the addition of IL-8 contributed to the increased survival of irradiated cancer cells (Fig. S2a and b). Although the level of hepatocyte growth factor (HGF) was also promoted and was testified (Fig. 2c; Fig. S2c), the addition of HGF failed to significantly improve cell recovery (Fig. S2d and e). An interruption of IL-8 signaling by a knockdown of IL-8 (Fig. S2f - h) or the addition of an IL-8 receptor antagonist (Fig. S3a; Fig. 2g and h) was found to restrict the survival of cancer cells promoted by CAFs after irradiation.

A Warburg effect was reported to create a more advantageous TME by increasing the tumor metastatic potential with a high level of lactate, and thereby enhance resistance to treatment [30, 31]. Critically, CAFs have emerged as a producer of a large amount of intermediate metabolites (e.g., lactate) that modulate glycolysis activity in HNSCC [6]. Intermediate metabolites produced by CAFs have been reported to enhance the proliferation of irradiated tumor cells [13]. Therefore, lactate production by CAFs in the supernatant was investigated. Although a difference was observed (Fig. S3b), the addition of a lactate production inhibitor failed to lead to an obvious impact on the survival of cancer cells which was consistent with previous research [13] (Fig. S3c and d).

### CAFs activate the NF-κB pathway in irradiated NPC cells

**Fig. 3:**
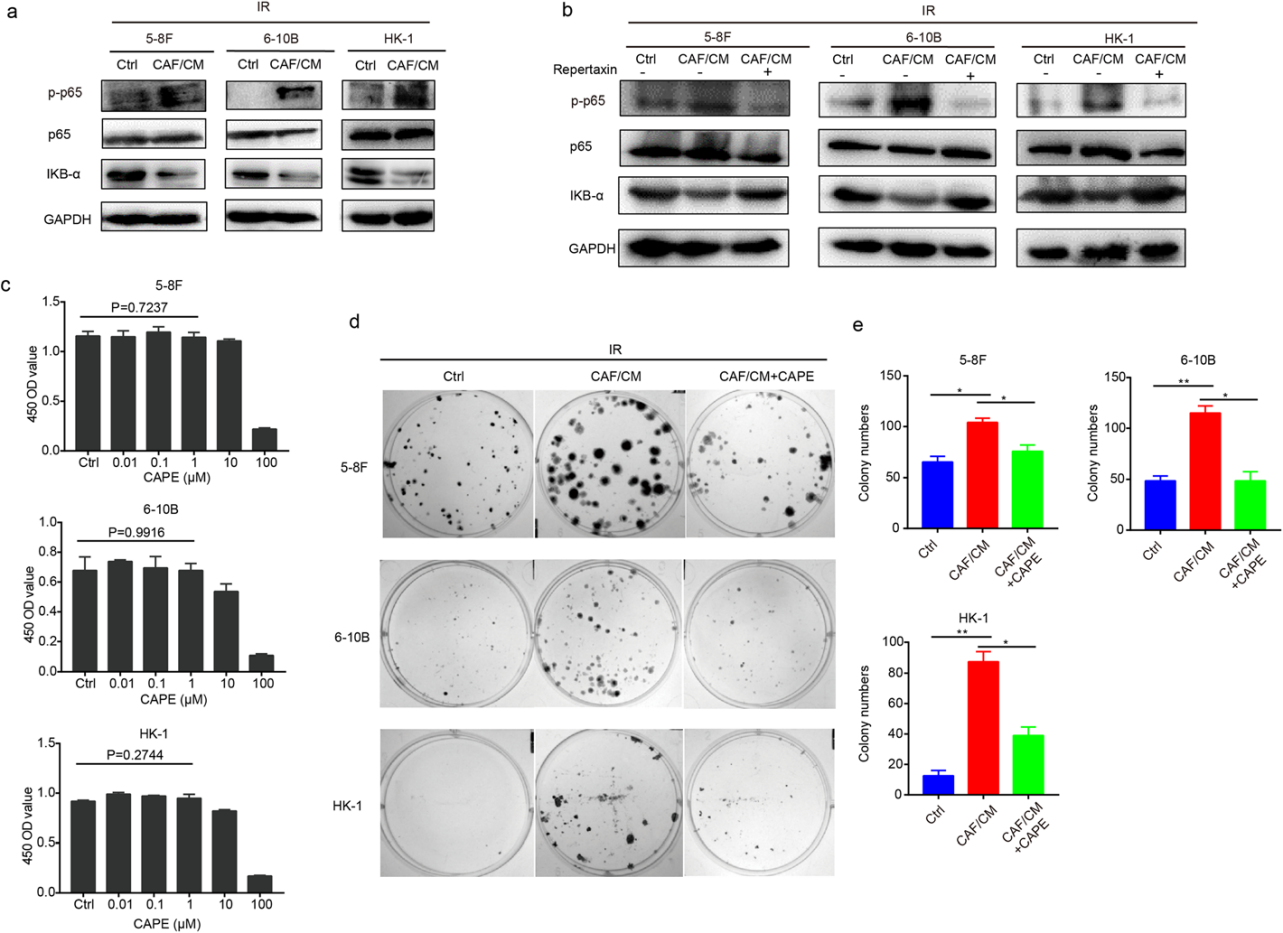
CAFs activated the NF-κB pathway in irradiated NPC cells. **a** CAFs activated the NF-κB pathway in irradiated NPC cells. **b** Treatment with an IL-8 receptor antagonist inhibited activation of the NF-κB pathway in irradiated NPC cells. **c** The proliferation of NPC cell lines was evaluated by a CCK-8 assay under a wide-range of concentrations of CAPE. **d** and **e** A blockade of the NF-κB pathway with CAPE (2 μM) inhibited the recovery of NPC cells after irradiation. * *P* < 0.05; ** *P* < 0.01; *** *P* < 0.001; **** *P* < 0.0001, ns, no significance

Tumor cells fed with CAF/CM exhibited increased proliferation and radioresistance properties after irradiation. To further uncover the underlying signaling pathways involved, an NPC tissue microarray and GSE48501 dataset containing radioresistant tissues and cell lines, respectively were analyzed using GSEA software. Given the circumstances, the NF-κB pathway was both substantially up-regulated in radio-resistant NPC tissues and cell lines (Fig. S4a - c). Differential expression genes of tissues were shown in supplementary material. Next, we established a radiation-resistant NPC 5-8F cell line and determined the sensitivity to irradiation compared to the parental cells (Fig. S4d and e). A transcription sequencing analysis was conducted and showed that the NF-κB pathway was also activated in 5-8F cells when acquiring radioresistance properties (Fig. S4f). In addition, protein levels of phosphorylated p65 involved in this pathway was also up-regulated (Fig. S4g). Consistently, the NF-κB pathway was activated with promoted protein levels of phosphorylated p65 subunit and decreased expression of IKB-α in tumor cells post-irradiation following the addition of CM from CAFs (Fig. 3a). Interruption of the IL-8 signaling pathway inhibited NF-κB signaling pathway activation with decrease of phosphorylated p65 protein expression in irradiated tumor cells (Fig. 3b). Following exposure to irradiation, the survival of cancer cells was augmented under stimulation with CAF/CM, which was reversed when an NF-κB pathway inhibitor was added at a concentration of 2 μM (Fig. 3c- e), indicating the requirement of the NF-κB pathway in promoting the recovery of NPC cells following irradiation.

### CAFs reduce DNA damage caused by irradiation

**Fig. 4:**
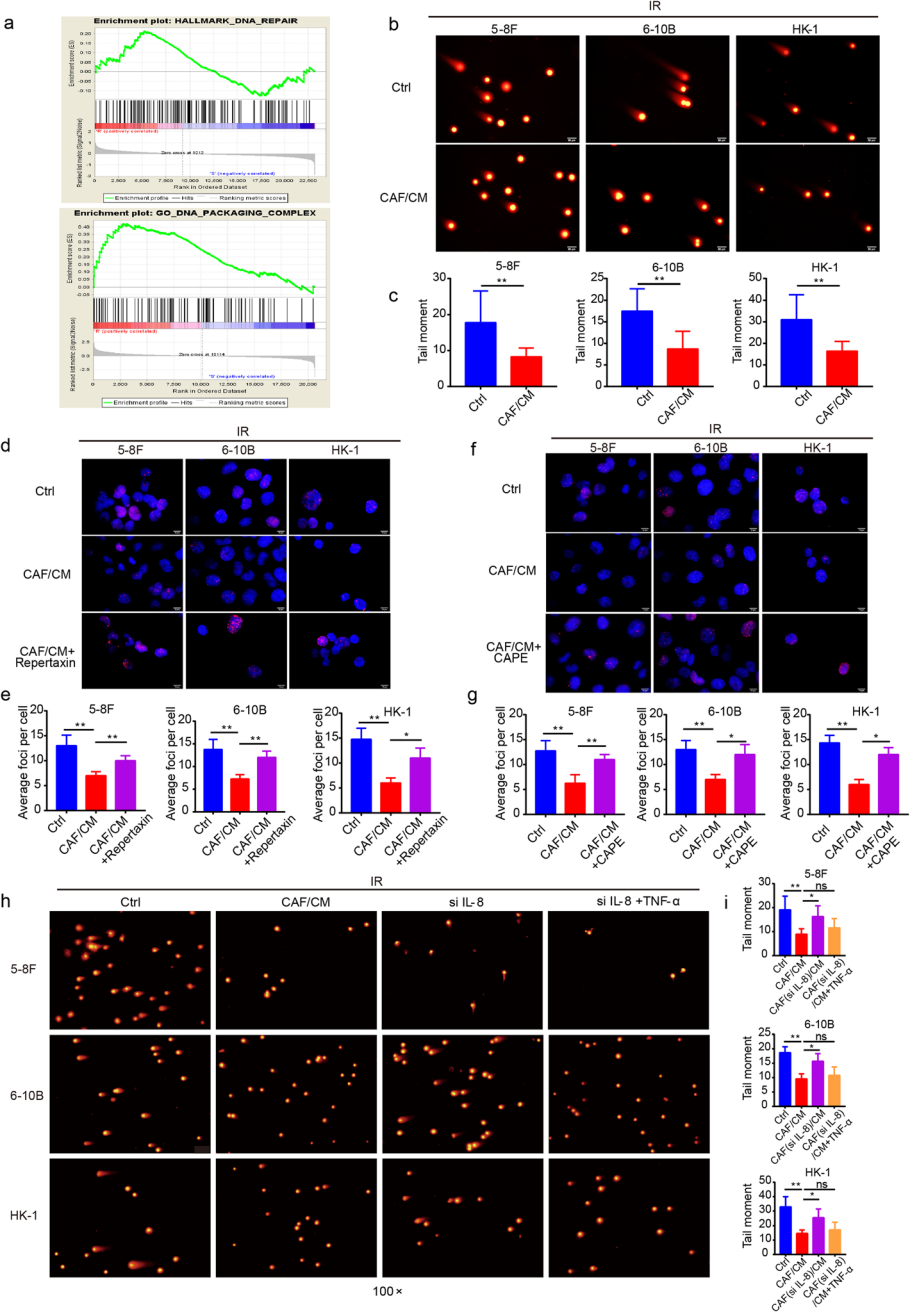
CAFs reduced the level of DNA damage in NPC cells after irradiation. **a** GSEA of GSE48501 cell microarray and tissue microarray were conducted and pathways related to DNA damage and repair were enriched. **b** and **c** Comet assay showed that CAFs reduced the level of DNA damage in irradiated tumor cells. **d** and **e** Interruption of the IL-8 signaling pathway increased the distribution of γ-H2AX foci post-irradiation. **f** and **g** A blockade of the NF-κB pathway increased γ-H2AX foci in irradiated NPC cells. **h** and **i** A blockade of the IL-8/NF-κB pathway increased the level of DNA damage in irradiated NPC cells. * *P* < 0.05; ** *P* < 0.01; *** *P* < 0.001; **** *P* < 0.0001, ns, no significance

Since irradiation is an effective therapeutic strategy, it mainly eliminates tumors by inducing DNA damage, including single strand and double strand brakes [32]. DNA damage repair was reported to be a frequently occurring event in radioresistant tumors [33]. The GSEA results revealed an enrichment of DNA repair-related signaling pathways in radioresistant NPC tissue (Fig. 4a). Therefore, we speculated that CAFs could have an impact on DNA damage repair to promote tumor growth following irradiation. The results of the comet assay demonstrated that cells stimulated with CAF/CM after irradiation had fewer and shorter tails (Fig. 4b and c), indicating the reduced level of DNA damage. Cultured with CAF/CM led to fewer γ-H2AX foci and an IL-8 blockade or NF-κB pathway blocking could restore the expression of γ-H2AX (Fig. 4d-g). Further, the activation of NF-κB pathway via TNF-α reduced the degree of DNA damage (Fig. 4h and i). All observations revealed that CAFs reduced the level of DNA damage via the IL-8/NF-κB signaling pathway in tumor cells following irradiation.

### Tranilast inhibits CAFs functionality

**Fig. 5:**
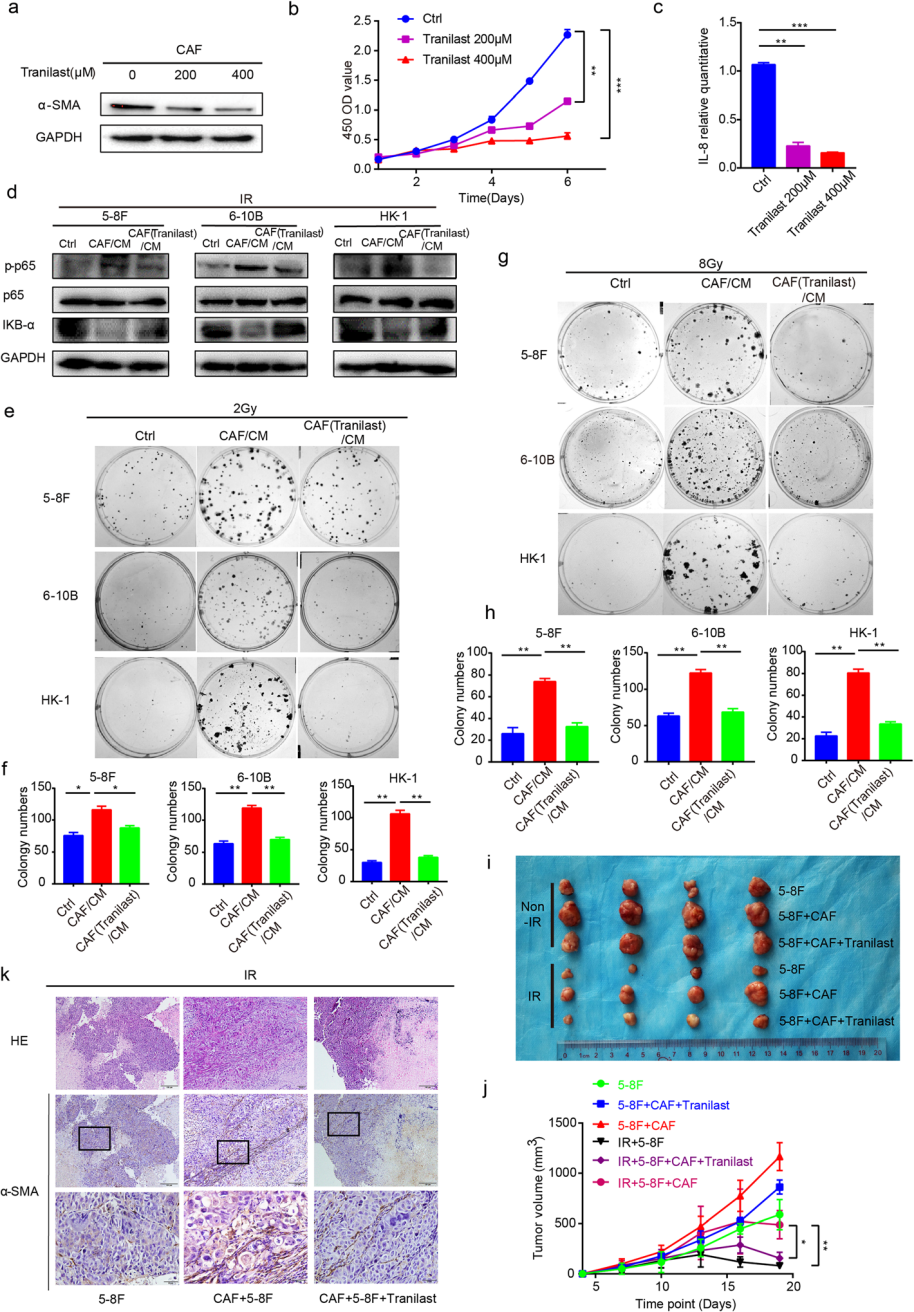
Tranilast treatment inhibited CAFs activity and functionality. **a** Treatment with Tranilast inhibited the activation of CAFs. **b** Tranilast inhibited the proliferation of CAFs. **c** Tranilast down-regulated IL-8 at the transcription level. **d** Tranilast restricted the activation of the NF-κB pathway in NPC cells. **e** and **f** Tranilast restricted the proliferation of NPC cells after irradiation. **g** and **h** Tranilast restored the radiosensitivity of NPC cells. **i** and **j** Tranilast inhibited the proliferation of irradiated NPC cells in vivo. **k** Represented images of HE staining and α-SMA staining of in vivo experiment were shown. * *P* < 0.05; ** *P* < 0.01; *** *P* < 0.001; **** *P* < 0.0001, ns, no significance

Accumulating evidence indicates that Tranilast is capable of inhibiting CAFs functionality [14]; however, little is known regarding whether Tranilast can impede the induction of radiation-resistance and attenuate CAF-induced NPC cell survival. Therefore, we first focused on the impact of Tranilast treatment on CAFs. As expected, α-SMA expression, a marker of CAFs activation, was significantly decreased following treatment with Tranilast (Fig. 5a). Moreover, Tranilast inhibited CAFs viability and proliferation in a concentration-dependent manner (Fig. 5b).

**Fig. 6:**
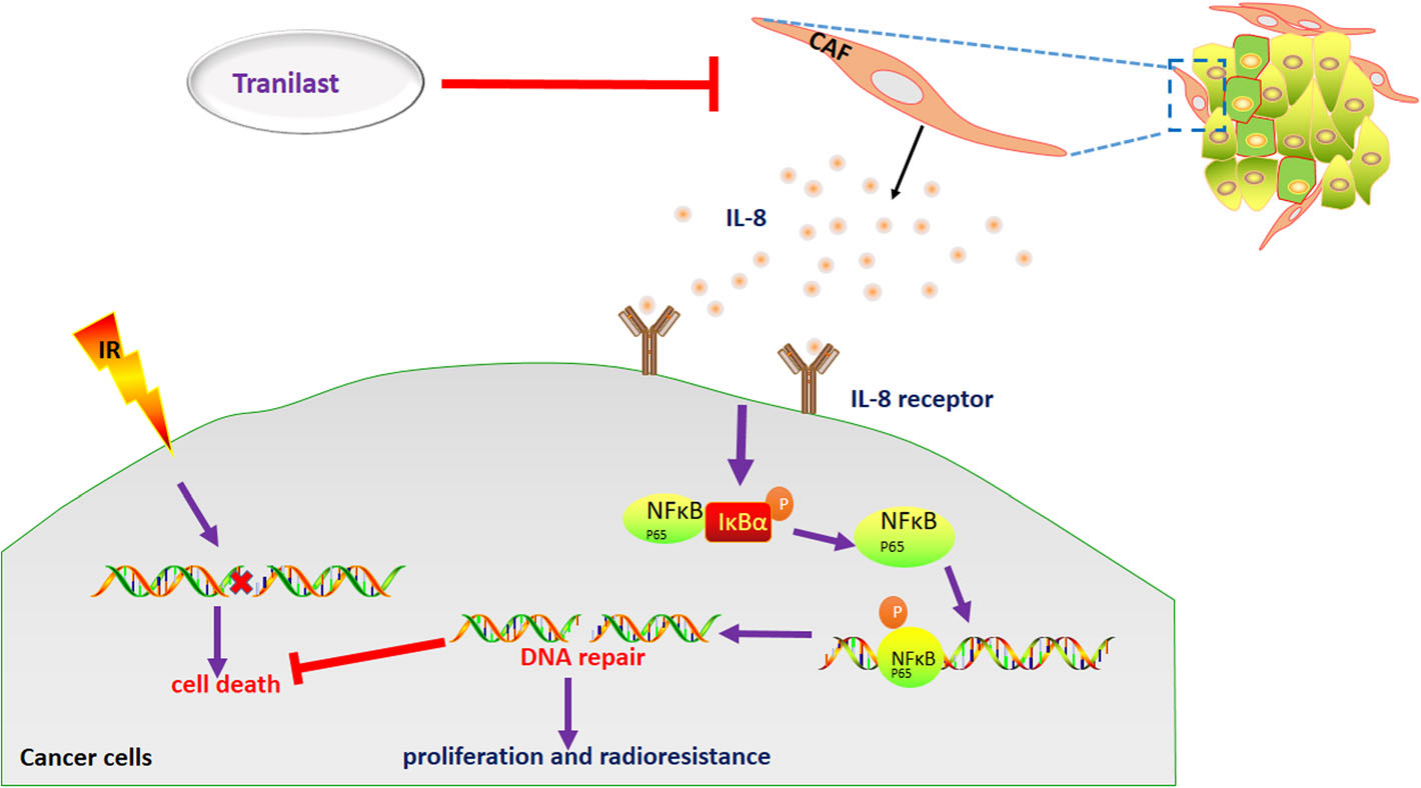
CAFs promoted the survival of irradiated NPC cells via the NF-κB pathway. Irradiation kills cancer cells via DNA damage. CAFs within TME promoted irradiated cancer cell survival by secretion of IL-8 to activate NF-κB signaling pathway, thus to enhance broken DNA repair and induced formation of radioresistance. Tranilast sensitized cancer cells to irradiation through the suppression of CAFs functionally, thus promoted irradiation-induced cell death

Functionally, we detected the level of IL-8 in CAFs treated with Tranilast. Similarly, the level of IL-8 mRNA was significantly down-regulated following an intervention with Tranilast compared to that of the control group (Fig. 5c). Moreover, Tranilast disrupted the activation of the NF-κB pathway via down-regulation of phosphorylated p65 protein in tumor cells (Fig. 5d) and resulted in a larger degree of DNA damage (Fig. S5a - d). Next, CM from CAFs previously treated with or without Tranilast were collected to culture cancer cells after irradiation with 2Gy. The group stimulated with CM from CAFs ever-treated with Tranilast revealed decreased survival of cells after irradiation when compared with the control group (Fig. 5e and f). Moreover, Tranilast also reversed the radioresistance characteristics induced by CAFs (Fig. 5g and h). In vivo, tumor cells mixed with CAFs were subcutaneously implanted into nude mice. After irradiation, tumors co-injected with CAFs grew faster than both co-injected Tranilast with CAFs group, and control group. This indicated that CAF plays a key role in promoting the development of irradiated tumors (Fig. 5i and j). Expression of α-SMA staining in CAF co-injected group was also higher than that in the above two groups (Fig. 5k; Fig. S5e). All of the above results suggest that Tranilast inhibits the functional activity of CAFs to inactivate the NF-κB pathway, thereby leading to increased cancer cell death and sensitization of NPC cells to irradiation (Fig. 6).

## Discussion

NPC is an EBV-related cancer, which is especially prevalent in Guangdong province, China [34]. Clinically, the challenges following relapse after irradiation are unavoidable and one of the primary reasons for the poor prognosis of cancer treatment [16, 35]. However, the potential mechanisms for tumor relapse are complex and remain poorly understood. NPC is a type of solid carcinoma with several elements of the TME. To date, the TME has gained increasing attention as a nutrient source required for cancer aggressiveness. Current research has mainly focused on the TME rather than only on the tumors, emphasizing the importance of the TME in tumor progression.

The TME is composed of cellular and non-cellular components and has been reported to promote tumor recurrence following irradiation [13, 36]. Of the plethora of cell types within the TME, CAFs are predominant, present in abundance and exhibiting distinct properties in numerous cancers [37]. In head and neck cancer, a high degree of CAF infiltration was reported to accelerate tumor progression via the regulation of metabolism activity [6]. Moreover, CAFs resident in NPC have been reported to promote tumor migration and invasion, thereby contribute to a poor prognosis. Despite these findings, studies involving CAFs and irradiation in NPC are inadequate. According to our findings, there was substantial CAF infiltration in the radioresistant NPC tissue compared with radiosensitive NPC tissue. Generally, tumors with abundant stromal components are usually stiff. This finding was consistent with the observation that patients with poor clinical efficacy usually present with stiff tumor [38]. Moreover, CAFs were found to both enhance survival and induce radioresistance in irradiated NPC cells, illustrating the multifunctionality of CAFs within the TME for NPC. As previously documented, CAFs modified the TME primarily through the continuous secretion of inflammatory cytokines essential for tumor proliferation, migration, invasion, metastasis and, resistance to treatment (e.g., vascular endothelial growth factor [VEGF] and HGF) [39–41]. Pathways that respond to irradiation, including the Wnt /β-catenin, β1-integrin, and P38 signaling pathways, have been identified [10, 42, 43]. In addition, IL-8 and HGF have been reported to be secreted by CAFs to promote HNSCC aggressiveness and were associated with worse prognosis [44, 45]. Based on our research, CAFs were found to enhance cell proliferation after irradiation via the secretion of IL-8 to trigger NF-κB activation with up-regulation of p-p65 in NPC cells. Disruption of NF-κB signaling impaired the recovery of tumor cells after irradiation promoted by CAFs.

In general, radiation can destroy DNA and result in damage ranging from nucleotide lesions to single- and double-strand breaks (SSBs and DSBs) [46], thereby killing tumor cells. The underlying mechanisms responsible for radioresistance are highly variable, among which include DNA damage repair [47, 48]. Recently, CAFs have been reported to assist cancer cell recovery from irradiation through autophagy, which is related to the DNA damage repair pathway [13]. Therefore, we hypothesized that CAFs could reduce the level of DNA damage to NPC cells following irradiation. Detection of the γ-H2AX protein that participated in the process of DNA damage [49, 50] showed that CAFs actually impaired tumor cell DNA damage. Moreover, NF-κB signaling was reported to engage in irradiation resistance in glioblastoma and pathogenesis of several cancer [51, 52]. Accordingly, we showed that a blockade of IL-8/NF-κB signaling interrupted NPC survival after irradiation, emphasizing the significance of the NF-κB pathway during the process of radioresistance.

Even though CAFs can have multiple cells-of-origin, such as stellate cells, mesenchymal stem cells, mesothelial cells and several other potential sources, resident fibroblasts are considered to be the most important source of CAFs [53]. In our study, CAFs were proved to promote irradiated tumor proliferation when co-injection with tumor cells in vivo. Similarly, it was reported that CAFs co-injected with tumor cells could be detected and persisted in tumor tissues [54, 55]. Nonetheless, it was also demonstrated by several studies that CAFs in xenograft tumors disappear shortly after co-injection [56]. We speculated an early enhancement of proliferation in tumor cells when co-injection with CAFs. This effect could persist for an indicated time once the critical signaling pathways were activated in tumor cells, even though injected CAFs gradually disappeared later. Insights into the mechanisms require further investigation.

Due to the predominant abundance within TME and the versatile properties essential for tumorigenesis, CAFs were considered to be a potential target cell type for therapeutic options. Previous studies have implied that Tranilast, a CAF inhibitor, could inhibit CAF proliferation and activation, as well as suppress the release of bioactive cytokines produced by CAFs [57]. Consistent with these reports, our data demonstrated that Tranilast inhibited CAF proliferation and reduced CAF activation as evidenced by lower α-SMA expression. Functionally, Tranilast impeded NPC cell growth after irradiation and restored the radiosensitivity of tumor cells against CAFs. Mechanically, Tranilast blocked the activation of NF-κB pathway and reversed the DNA damage induced by irradiation. All data indicate that Tranilast may be a promising inhibitor against CAFs to accelerate NPC elimination after irradiation. And the potential function of Tranilast to sensitize NPC to irradiation needs further research in the future.

## Conclusions

In summary, our findings revealed the importance of CAFs in NPC progression following irradiation by enhancing the survival of tumor cells, which promotes radioresistance. Mechanistically, IL-8 secretion by CAFs activated the NF-κB pathway in NPC, thus reducing the level of DNA damage caused by irradiation. Importantly, treatment with Tranilast inhibited CAF functionality and sensitized the tumor cells to irradiation; however, additional clinical samples are warranted to further validate the relationship among CAFs, radioresistance in tumor cells.

## Supplementary Information

**Additional file 1: Table1.** Patient and tumor characteristics.

**Additional file 2. **List of differential expression genes.

**Additional file 3: Figure S1.** Infiltration of CAFs in NPC tissue. a and b Expression of FAP was higher in radioresistant NPC than radiosensitive NPC tissue. C Represented images of α-SMA and FAP staining of one NPC donor tissue used for primary culture were shown. * *P* < 0.05; ** *P* < 0.01; *** *P* < 0.001; **** *P* < 0.0001, ns, no significance.

**Additional file 4: Figure S2.** CAFs promoted the recovery of irradiated tumor cells via IL-8. a and b IL-8 promoted the recovery of tumor cells after irradiation. c High levels of HGF were verified in CAFs. d and e HGF failed to significantly promote the recovery of tumor cells after irradiation. f The efficiency of knocking down IL-8 was verified by real-time PCR. g and h Knock-down of IL-8 in CAFs inhibited the survival of NPC cells after irradiation. * *P* < 0.05; ** *P* < 0.01; *** *P* < 0.001; **** *P* < 0.0001, ns, no significance.

**Additional file 5: Figure S3.** CAF promoted the survival of irradiated tumor cells. a The proliferation of NPC cell lines was evaluated by CCK-8 assay under a diverse range of Repertaxin concentrations. b CAFs produced a higher amount of lactate than NFs. c and d Disruption of lactate production with a lactate inhibitor (DCA) failed to promote the survival of irradiated tumor cells. * *P* < 0.05; ** *P* < 0.01; *** *P* < 0.001; **** *P* < 0.0001, ns, no significance.

**Additional file 6: Figure S4.** The NF-κB pathway was activated in radioresistant NPC cells and tissues. a and b The NF-κB pathway was substantially up-regulated in radioresistant cells and tissues. c Heatmap of differentially regulated genes in the tissue microarray was shown. d and e The 5-8F IRR cell line was established and a survival curve at different dose was created. f and g The NF-κB pathway was significantly activated in the 5-8F IRR cell line, which was verified by western blot. * *P* < 0.05; ** *P* < 0.01; *** *P* < 0.001; **** *P* < 0.0001, ns, no significance.

**Additional file 7: Figure S5.** Tranilast restored irradiation-induced DNA damage in irradiated tumor cells. a and b Comet assay showed that Tranilast treatment reversed the DNA repair promoted by CAFs. c and d Tranilast restored the distribution of γ-H2AX foci in irradiated tumor cells. e Analysis of α-SMA staining of in vivo experiment.* *P* < 0.05; ** *P* < 0.01; *** *P* < 0.001; **** *P* < 0.0001, ns, no significance.

## Data Availability

All data that can prove the conclusion of this article are included in the article.

## Abbreviations

NPC: Nasopharyngeal carcinoma
HNSCC: Head and neck squamous cell carcinoma
EBV: Epstein-Barr virus
CAF: Cancer-associated fibroblast
NF: Normal fibroblast
RSI: Radiosensitivity index
IRR: Irradiation resistant
IR: Irradiation
α-SMA: Alpha smooth muscle Actin
FAP: Fibroblast activated protein
VEGF: Vascular endothelial growth factor
NF-κB: Nuclear factor kappa-B
IL-8: Interleukin 8
HGF: Hepatocyte growth factor
RT-qPCR: Quantitative real-time polymerase chain reaction
GSEA: Gene set enrichment analysis
DSBs: Double-strand breaks
SSBs: Single-strand breaks
