# Correction to: Cancer-associated fibroblasts promote the survival of irradiated nasopharyngeal carcinoma cells via the NF-κB pathway

**DOI:** 10.1186/s13046-021-01914-w

**Published:** 2021-03-22

**Authors:** Weiqiang Huang, Longshan Zhang, Mi Yang, Xixi Wu, Xiaoqing Wang, Wenqi Huang, Lu Yuan, Hua Pan, Yin Wang, Zici Wang, Yuting Wu, Jihong Huang, Huazhen Liang, Shaoqun Li, Liwei Liao, Laiyu Liu, Jian Guan

**Affiliations:** 1 Department of Radiation Oncology, Nanfang Hospital, Southern Medical University, Guangzhou, Guangdong China; 2 Chronic Airways Diseases Laboratory, Department of Respiratory and Critical Care Medicine, Nanfang Hospital, Southern Medical University, Guangzhou, Guangdong China; 3 Department of Obstetrics and Gynecology, The First Affiliated Hospital of Guangzhou Medical University, Guangzhou, Guangdong China; 4 Department of Oncology, Maoming People’s Hospital, Maoming, Guangdong China; 5 Department of Radiation Oncology, Guangdong 999 Brain Hospital, Guangzhou, Guangdong China

**Correction to: J Exp Clin Cancer Res 40, 87 (2021)**

**https://doi.org/10.1186/s13046-021-01878-x**

Following publication of the original article [1], the authors and publisher identified errors in the typesetting of the supplementary material. Whilst the supplementary file captions were correct, the supplementary material files had been ordered incorrectly within the article.

The original article has been corrected.
