# Supplementary material for: Cancer-associated fibroblasts promote the survival of irradiated nasopharyngeal carcinoma cells via the NF-κB pathway
Source: J Exp Clin Cancer Res. 2021 Mar 1;40:87. doi: 10.1186/s13046-021-01878-x (PMC7923322; doi:10.1186/s13046-021-01878-x)
Supplement: Supplementary file 1 — Additional file 1: Table1. Patient and tumor characteristics. [file 13046_2021_1878_MOESM1_ESM.docx]

**Table1 Patient and tumor characteristics**

|  | CASE 1 | CASE 2 | CASE 3 | CASE 4 |
| --- | --- | --- | --- | --- |
| Sex | Male | Male | Female | Female |
| Age | 50 | 39 | 43 | 34 |
| UICC T stage | rT0N1M0 | rT2N0M0 | rT4N0M0 | rT3N0M0 |
| Previous treatment regimen | CRT^*^ | Neoadjuvant Chemo +CRT | Neoadjuvant Chemo +CRT | Neoadjuvant Chemo +CRT |
| Pathology | Keratinised squamous carcinoma | Undifferentiated non keratinised | Undifferentiated non keratinised | Undifferentiated non keratinised |
| Plasma EBV-DNA | - | < 500 copies/mL | > 500 copies/mL | < 500 copies/mL |

UICC = Union for International Cancer Control (8th Edition); CRT = concurrent chemoradiotherapy; Chemo = chemotherapy; EBV = Epsteine-Barr virus.

*: Previous irradiation dose/Fraction: PGTV: 6996cGy/33F; PCTV1:6303cGy/33F; PCTV2:5940cGy/30F
