# Supplementary figures and images for: Cancer-associated fibroblasts promote the survival of irradiated nasopharyngeal carcinoma cells via the NF-κB pathway
Source: J Exp Clin Cancer Res. 2021 Mar 1;40:87. doi: 10.1186/s13046-021-01878-x (PMC7923322; doi:10.1186/s13046-021-01878-x)

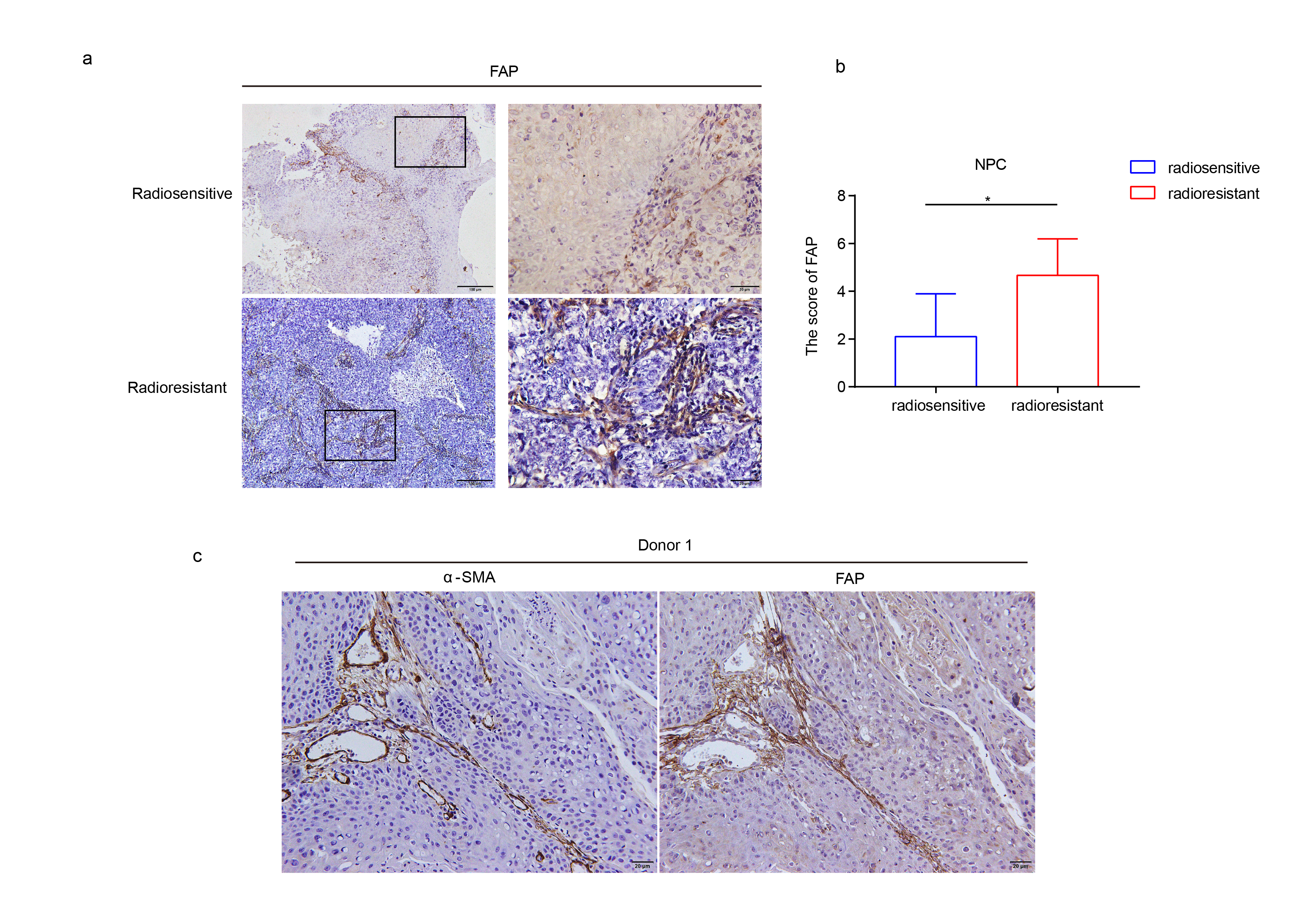

Supplement: Supplementary file 3 — Additional file 3: Figure S1. Infiltration of CAFs in NPC tissue. a and b Expression of FAP was higher in radioresistant NPC than radiosensitive NPC tissue. C Represented images of α-SMA and FAP staining of one NPC donor tissue used for primary culture were shown. * P < 0.05; ** P < 0.01; *** P < 0.001; **** P < 0.0001, ns, no significance. [file 13046_2021_1878_MOESM3_ESM.tif]

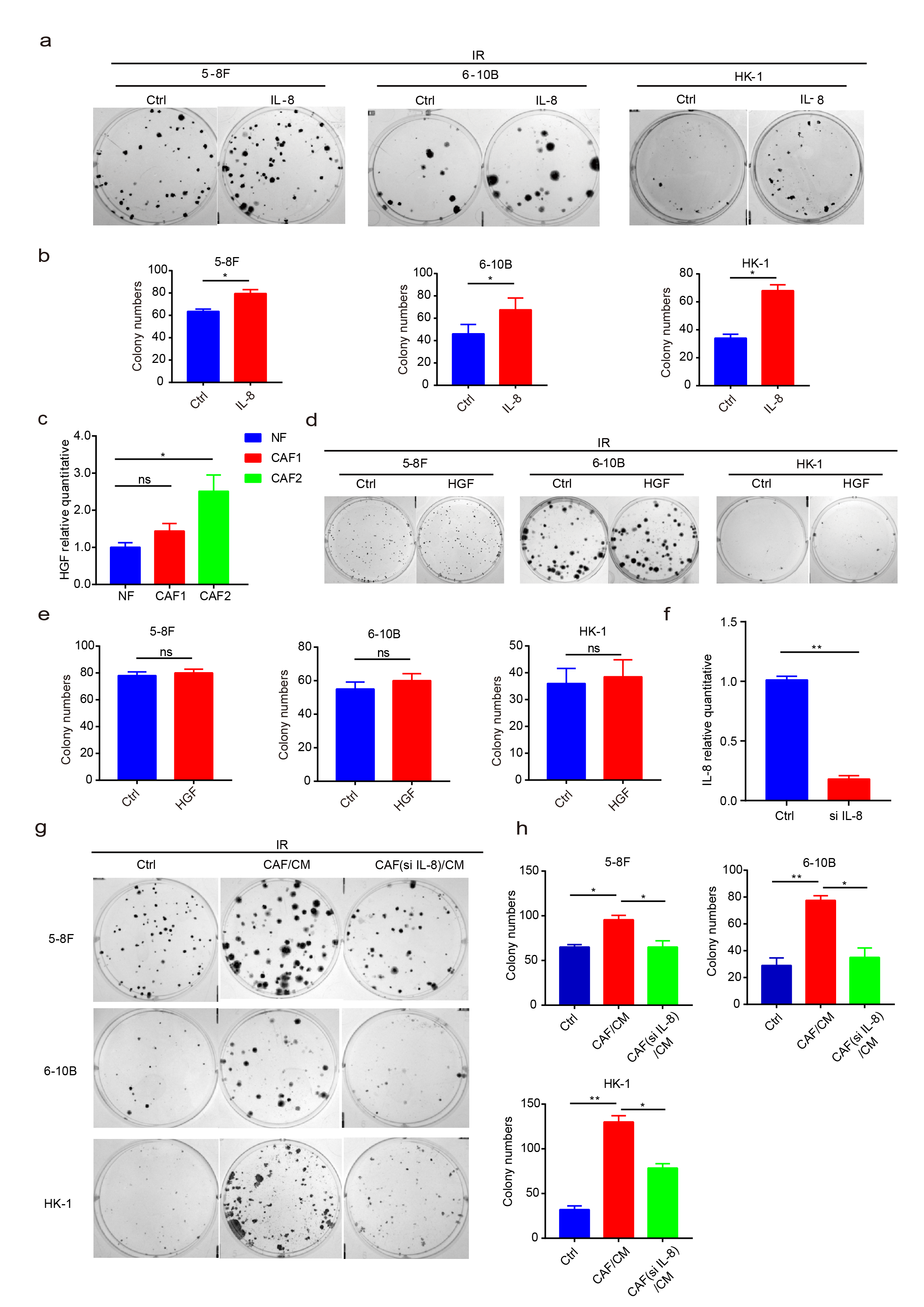

Supplement: Supplementary file 4 — Additional file 4: Figure S2. CAFs promoted the recovery of irradiated tumor cells via IL-8. a and b IL-8 promoted the recovery of tumor cells after irradiation. c High levels of HGF were verified in CAFs. d and e HGF failed to significantly promote the recovery of tumor cells after irradiation. f The efficiency of knocking down IL-8 was verified by real-time PCR. g and h Knock-down of IL-8 in CAFs inhibited the survival of NPC cells after irradiation. * P < 0.05; ** P < 0.01; *** P < 0.001; **** P < 0.0001, ns, no significance. [file 13046_2021_1878_MOESM4_ESM.tif]

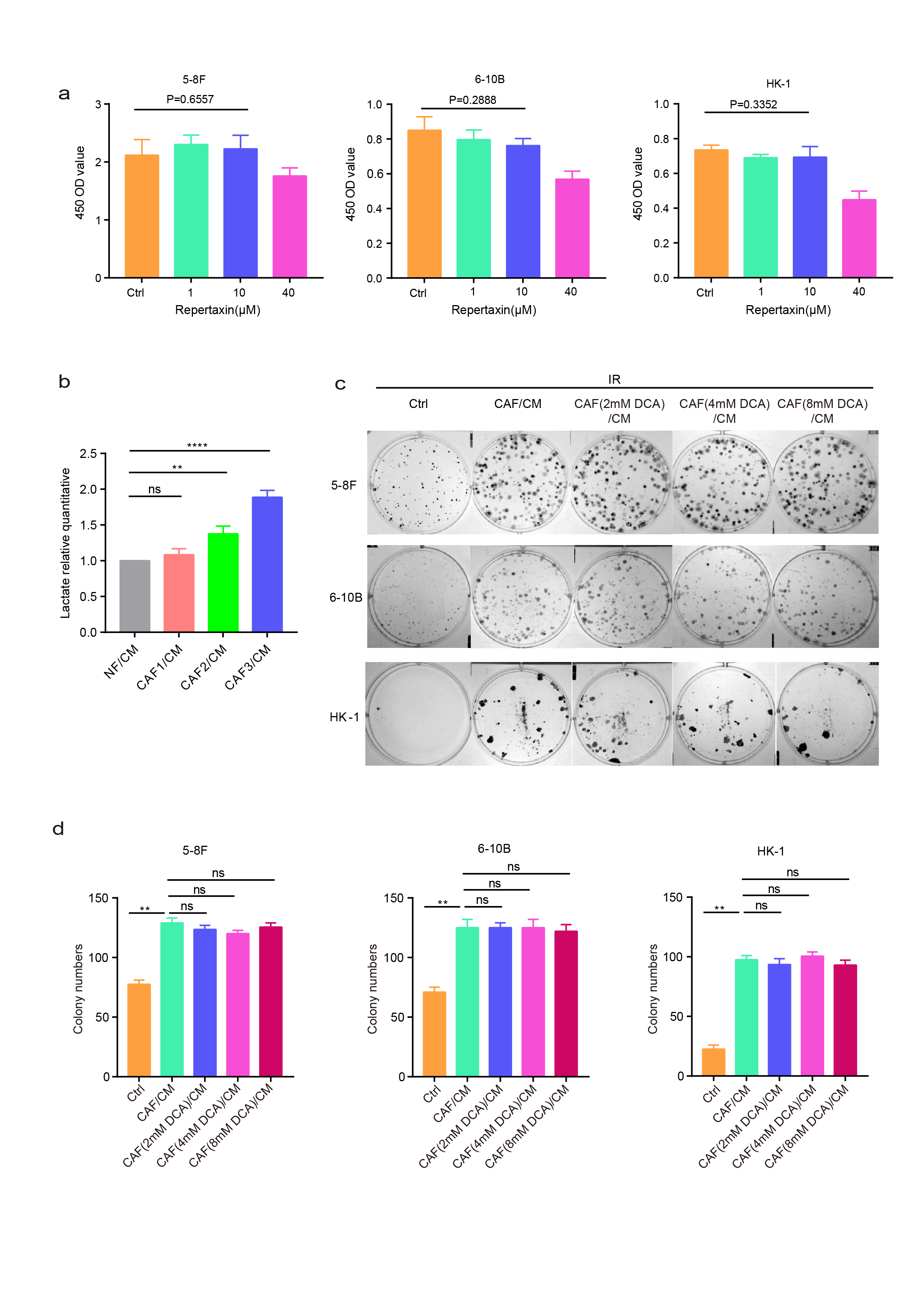

Supplement: Supplementary file 5 — Additional file 5: Figure S3. CAF promoted the survival of irradiated tumor cells. a The proliferation of NPC cell lines was evaluated by CCK-8 assay under a diverse range of Repertaxin concentrations. b CAFs produced a higher amount of lactate than NFs. c and d Disruption of lactate production with a lactate inhibitor (DCA) failed to promote the survival of irradiated tumor cells. * P < 0.05; ** P < 0.01; *** P < 0.001; **** P < 0.0001, ns, no significance. [file 13046_2021_1878_MOESM5_ESM.tif]

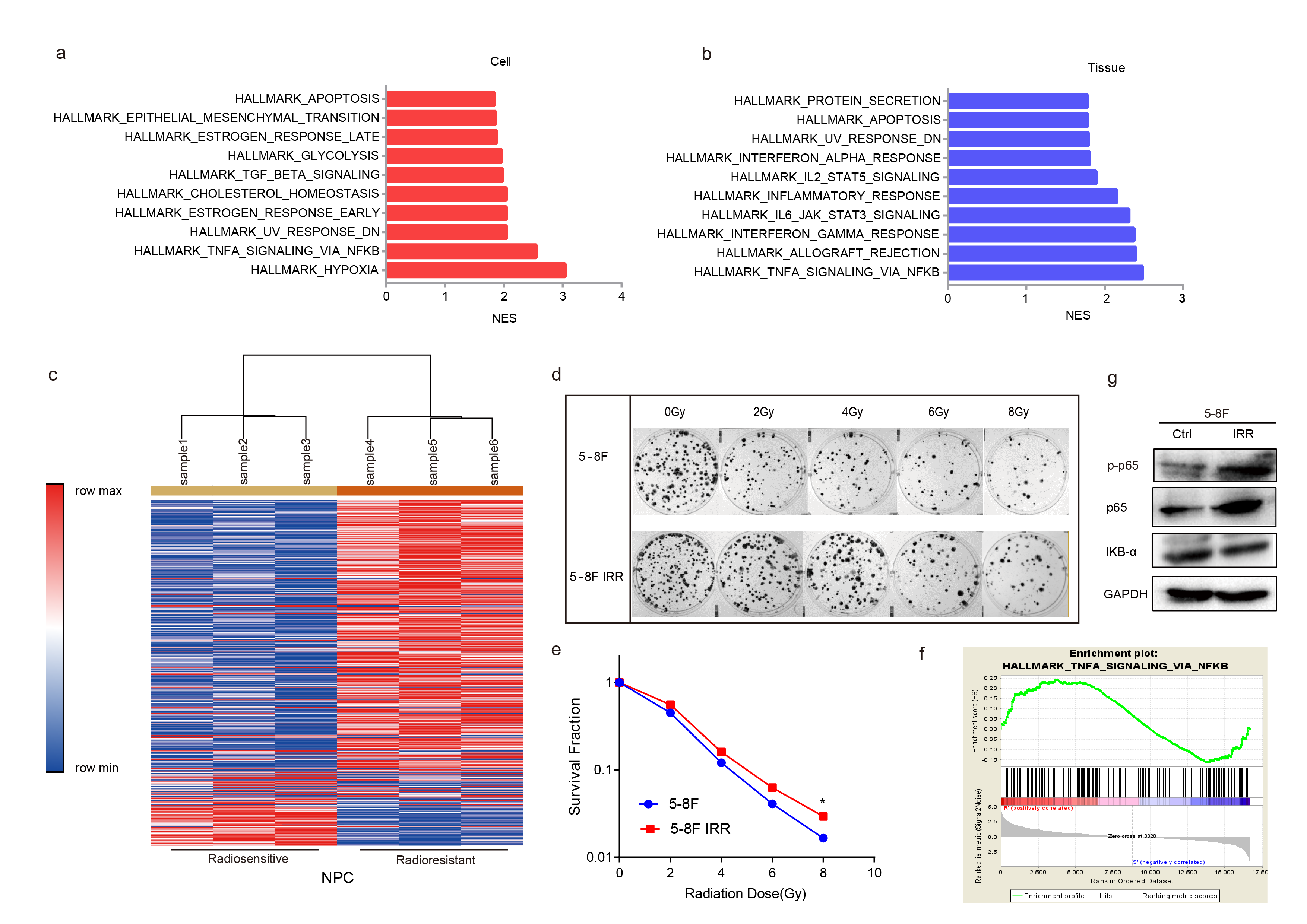

Supplement: Supplementary file 6 — Additional file 6: Figure S4. The NF-κB pathway was activated in radioresistant NPC cells and tissues. a and b The NF-κB pathway was substantially up-regulated in radioresistant cells and tissues. c Heatmap of differentially regulated genes in the tissue microarray was shown. d and e The 5-8F IRR cell line was established and a survival curve at different dose was created. f and g The NF-κB pathway was significantly activated in the 5-8F IRR cell line, which was verified by western blot. * P < 0.05; ** P < 0.01; *** P < 0.001; **** P < 0.0001, ns, no significance. [file 13046_2021_1878_MOESM6_ESM.tif]

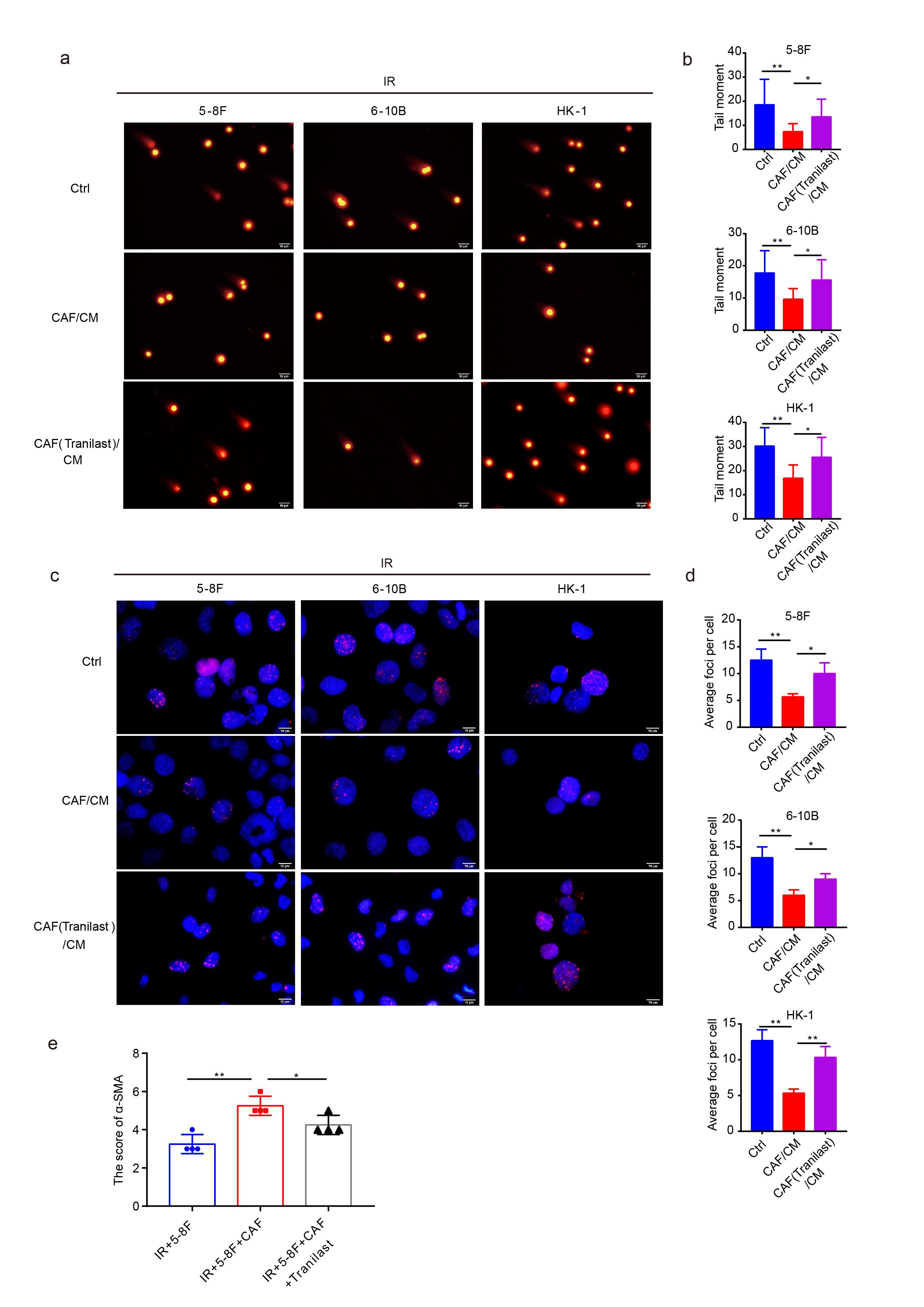

Supplement: Supplementary file 7 — Additional file 7: Figure S5. Tranilast restored irradiation-induced DNA damage in irradiated tumor cells. a and b Comet assay showed that Tranilast treatment reversed the DNA repair promoted by CAFs. c and d Tranilast restored the distribution of γ-H2AX foci in irradiated tumor cells. e Analysis of α-SMA staining of in vivo experiment.* P < 0.05; ** P < 0.01; *** P < 0.001; **** P < 0.0001, ns, no significance. [file 13046_2021_1878_MOESM7_ESM.tif]
